# Comparing Pandemic to Seasonal Influenza Mortality: Moderate Impact Overall but High Mortality in Young Children

**DOI:** 10.1371/journal.pone.0031197

**Published:** 2012-02-03

**Authors:** Cees C. van den Wijngaard, Liselotte van Asten, Marion P. G. Koopmans, Wilfrid van Pelt, Nico J. D. Nagelkerke, Cornelia C. H. Wielders, Alies van Lier, Wim van der Hoek, Adam Meijer, Gé A. Donker, Frederika Dijkstra, Carel Harmsen, Marianne A. B. van der Sande, Mirjam Kretzschmar

**Affiliations:** 1 National Institute for Public Health and the Environment, Center for Infectious Disease Control, Bilthoven, The Netherlands; 2 Erasmus Medical Center, Rotterdam, The Netherlands; 3 United Arab Emirates University, Al-Ain, United Arab Emirates; 4 NIVEL, Netherlands Institute of Health Services Research, Utrecht, The Netherlands; 5 Statistics Netherlands, The Hague, The Netherlands; 6 Julius Centre for Health Sciences & Primary Care, University Medical Centre, Utrecht, The Netherlands; Massey University, New Zealand

## Abstract

**Background:**

We assessed the severity of the 2009 influenza pandemic by comparing pandemic mortality to seasonal influenza mortality. However, reported pandemic deaths were laboratory-confirmed – and thus an underestimation – whereas seasonal influenza mortality is often more inclusively estimated. For a valid comparison, our study used the same statistical methodology and data types to estimate pandemic and seasonal influenza mortality.

**Methods and Findings:**

We used data on all-cause mortality (1999–2010, 100% coverage, 16.5 million Dutch population) and influenza-like-illness (ILI) incidence (0.8% coverage). Data was aggregated by week and age category. Using generalized estimating equation regression models, we attributed mortality to influenza by associating mortality with ILI-incidence, while adjusting for annual shifts in association. We also adjusted for respiratory syncytial virus, hot/cold weather, other seasonal factors and autocorrelation. For the 2009 pandemic season, we estimated 612 (range 266–958) influenza-attributed deaths; for seasonal influenza 1,956 (range 0–3,990). 15,845 years-of-life-lost were estimated for the pandemic; for an average seasonal epidemic 17,908. For 0–4 yrs of age the number of influenza-attributed deaths during the pandemic were higher than in any seasonal epidemic; 77 deaths (range 61–93) compared to 16 deaths (range 0–45). The ≥75 yrs of age showed a far below average number of deaths. Using pneumonia/influenza and respiratory/cardiovascular instead of all-cause deaths consistently resulted in relatively low total pandemic mortality, combined with high impact in the youngest age category.

**Conclusion:**

The pandemic had an overall moderate impact on mortality compared to 10 preceding seasonal epidemics, with higher mortality in young children and low mortality in the elderly. This resulted in a total number of pandemic deaths far below the average for seasonal influenza, and a total number of years-of-life-lost somewhat below average. Comparing pandemic and seasonal influenza mortality as in our study will help assessing the worldwide impact of the 2009 pandemic.

## Introduction

The 2009 influenza A/H1N1 pandemic has led to major additional surveillance and control efforts by public health authorities worldwide. Assessing the severity of the pandemic has been complicated. A concrete measure of severity would be the number of deaths (mortality) and years-of-life-lost due to the pandemic compared to the numbers caused by seasonal epidemics. However, the numbers of pandemic deaths reported to WHO [1], [2] were only those that were laboratory-confirmed and thus underestimate true numbers. These laboratory-confirmed figures cannot, therefore, be directly compared to seasonal influenza mortality estimates which – other than the pandemic deaths reported to WHO – are often based on statistical attribution of all-cause mortality to influenza [3], [4], [5]. To properly compare the 2009 pandemic mortality to seasonal influenza, the same statistical methodology should be used.

Another methodological issue is that young children seem to have been disproportionately affected by the pandemic, whereas the elderly have been relatively spared [6], [7], [8]. As years-of-life-lost decrease with increasing age of death, a proper comparison between pandemic and seasonal influenza should take into account the age distribution of fatal cases.

Two studies have estimated age-specific pandemic and seasonal influenza mortality for the US [9] and Australia [10], by attributing all-cause mortality to influenza if it deviated from a seasonal baseline, similar to earlier studies that estimated seasonal influenza mortality [3], [4], [5]. This approach assumes that all mortality deviations from the seasonal baseline during influenza epidemics are due to influenza. An alternative way to attribute all-cause mortality to influenza is using the modeled association of all-cause mortality and influenza activity in the population. In the Netherlands and many other countries, fluctuations of influenza activity in the population are reflected by the weekly influenza-like-illness (ILI) incidence as available from a sentinel network of general practitioners [11].

Nevertheless, associating all-cause mortality to ILI-incidence can be complicated by annual heterogeneity in the association between mortality and ILI. In an earlier study we already reported that such annual heterogeneity in association is likely to be due to differences in severity per ILI case driven by viral factors such as dominant (sub)types (influenza A(H1N1), A(H3N2) or B) and/or antigenic characteristics [12], but some heterogeneities may also be caused by media attention and public anxiety. This problem can be solved by allowing the modeled association of mortality with ILI to vary among years [12].

In this study we compared the 2009 pandemic mortality to the 10 preceding seasonal epidemics in the Netherlands (population size 16.5 million) by attributing age-specific all-cause mortality to influenza in 1999–2010, using the modeled association of weekly all-cause mortality with ILI-incidence. We also calculated years-of-life-lost from the age-specific influenza mortality estimates.

## Methods

### Mortality and influenza surveillance data

We obtained weekly mortality data from Statistics Netherlands (100% coverage) for the period 1999–2010, aggregated by week and age category (0–4, 5–24, 25–44, 45–64, 65–74 and ≥75 yrs of age). In addition to all-cause mortality, physician-reported primary cause of death data were available for analysis. Weekly ILI-incidence data for 1999–2010 were obtained from a sentinel network of general practitioners (GP-ILI data, 0.8% coverage) [11]. See Figure S1 for the time series of mortality by age category and ILI incidence. For analysis, successive years were defined to start on July 1^st^ and to end on June 30^th^, since influenza epidemics normally occur around wintertime.

### Data on laboratory detections

To adjust for possible mortality caused by pathogens other than influenza, we also investigated total weekly counts of RSV, as reported by a routine laboratory surveillance system (the Weekly Diagnostic Laboratory Reports of the Dutch Working Group on Clinical Virology, which covers 38–73% of viral diagnostics in the Netherlands; number of laboratory submissions (denominator) and patient age were not available [13]). As RSV is known to cause severe morbidity, especially in young children but also in adults [14], [15], it might affect estimates of influenza-associated mortality. During the pandemic, substantially more RSV infections were detected than in earlier years, probably due to increased requests for RSV diagnostics to exclude influenza. Such an artifact could bias estimates of the association of RSV with mortality. Therefore, we adjusted the RSV data during the pandemic downward by dividing the counts in 2009/2010 by a factor that reduces the total annual count to the mean annual count during the 4 years before 2009/2010. From the same laboratory network, weekly counts of influenza detections were also available as an alternative measure for influenza activity (instead of ILI-incidence) in a sensitivity analysis.

### Temperature data

Another contributor to (excess) mortality can be weather conditions, such as hot and/or cold temperatures [16], [17]. Therefore, we also calculated the weekly mean temperature from reported daily mean temperatures for 1999–2010 (www.knmi.nl) and defined weight variables for cold or warm weeks as the number of degrees Celsius below 0°C and above 17°C respectively.

### Regression analyses

Similar to [12], we used additive generalized estimating equation (GEE) [18] models with a Poisson distribution to attribute all-cause mortality to ILI-incidence. We decided to use the overall (instead of age-specific) ILI-incidence, because the coverage in the GP-ILI-sentinel may have been insufficient for some of the age categories included in the study. Thus we considered the overall ILI-incidence as a measure of influenza activity on the national level, associated with all-cause mortality by age category. As fluctuations in ILI-incidence may to some extent reflect activity of pathogens other than influenza, we only included the ILI-incidence within a −3/+3 week window around influenza epidemic episodes (defined as two or more successive weeks that the overall weekly ILI-incidence was above the threshold for influenza epidemics; i.e. 5.1 per 10,000 population [11]); in other weeks the ILI incidence was truncated at zero. First, we regressed mortality time series – separately for each age category – on (lagged) overall ILI-incidence, using a generalized linear model with a Poisson distribution and an identity link. To correct for mortality due to seasonal factors other than influenza, we started with a constant basic mortality level plus sine and cosine variables (sine(k2πweek/52) and cosine(k2πweek/52), k = 1,2,3,4). We also added the temperature variables as covariables to correct for mortality due to hot (mean daily temperature>17°C) and/or cold (mean daily temperature<0°C) weather conditions. We then added the ILI-incidence and the RSV counts to the model, selecting the lagged ILI and RSV variable that contributed most to the model fit (-4 up to +2 week lags were considered for inclusion; e.g., in step 1, ILI was included with a 1-week lag if that exhibited a better model fit than all other ILI/RSV-lag combinations, assessed with Akaike's information criterion [19]). We included lagged GP-ILI-incidence and the RSV counts in the model only once (i.e. for one lag). We used year-specific ILI regression coefficients as described in [12] so that the modeled association of mortality with ILI can vary among years. Essentially, these year-specific ILI regression coefficients are scaling factors for the number of deaths associated with a one-case increase in ILI-incidence per 10,000 population. Finally, we used GEEs [18] to correct the model outcomes for autocorrelation between observations. See Appendix S1 for details on the regression models.

We then used the model coefficients to estimate the weekly number of ILI-attributed deaths by age category. Based upon these weekly estimates of influenza-attributed deaths, we calculated the annual influenza-attributed numbers of deaths, scaled to the 2009 population. Finally, we compared the pandemic mortality to the influenza mortality in earlier years.

We also performed a sensitivity analysis to evaluate the impact of our model choices on the influenza-attributed mortality; we evaluated the impact of in- or excluding RSV or the hot/cold weather variables in the model, using ILI-incidence vs influenza detections as a measure of influenza activity, and using pneumonia/influenza (ICD-10 codes J09-J18) or respiratory/cardiovascular (J00-J99 and I00-I99) deaths as explained variables instead of all-cause deaths.

### Years-of-life-lost

We calculated the years-of-life-lost (YLL) [20] by multiplying each death with the residual life expectancy of its age group, which was derived from 2009 Dutch mortality data, as described in [21]. The residual life expectancy per age group was weighted by the average age of death in that group (Table 1).

**Table 1:** Life expectancy estimates in the Netherlands used for calculation of years-of-life-lost (YLL).

Age group	Average age at death in 2009 (yrs)	Life expectancy in 2009 (yrs)
0–4	0.62	80.26
5–24	17.93	63.23
25–44	38.13	43.44
45–64	57.63	25.33
65–74	70.43	14.93
≥75	85.65	5.52

## Results

### Regression analysis

**Figure 1:**
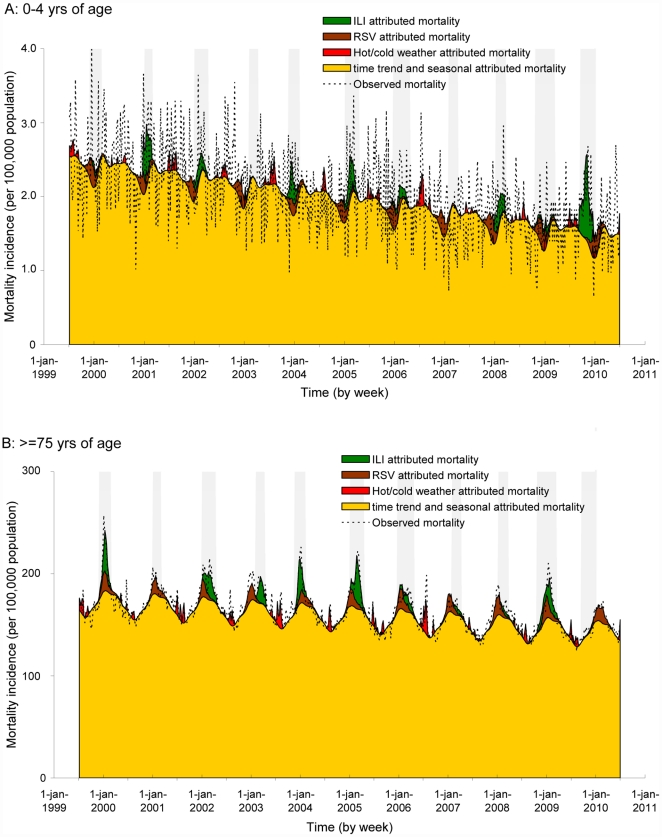
Weekly estimates for ILI-attributed mortality for the (a) 0–4 and (b) ≥75 yrs of age (data for other age categories not shown). For these estimates we used regression models with annual ILI-coefficients, corrected for RSV activity, hot/cold weather, seasonal factors and a time trend. The green colored areas reflect the estimated ILI-attributed mortality, and the black dotted lines present the actually observed weekly mortality; the orange colored areas reflect the estimated basis seasonal mortality, corrected for a time trend; the brown and red colored areas reflect mortality attributed to RSV and hot/cold weather conditions respectively. The grey-shaded bars on the background of the graphs indicate influenza epidemic episodes (defined as −3/+3 weeks around successive weeks with an overall ILI-incidence ≥5.1/10,000 population); only ILI-incidence in these episodes was included in the models. The strong downward timetrend in 0–4 yrs of age seems mainly due to a decrease of 39% in neonatal deaths in the last decade as reported by the national perinatal audit [45].

Fitting the regression models with annual ILI-coefficients to our data showed heterogeneity in the influenza-attributed mortality incidence both among years, and among age categories. Figure 1a–b illustrates this heterogeneity for the 0–4 and ≥75 yrs of age; for the 0–4 yrs of age there is a sharp elevation in the influenza-attributed mortality during the pandemic, whereas for the elderly this mortality is minimal compared to earlier years.

In the models, for all age categories the (lagged) ILI-incidence was included as explanatory variable for all-cause mortality. The annual ILI regression coefficients showed significant heterogeneity between years for all age categories (F-test, P≤0.0001). Some of the annual ILI regression coefficients estimates were below zero – reflecting the mild influenza impact in those years, as also observed in [12] – and were truncated at zero.

### Influenza-attributed mortality and years-of-life-lost

Table 2 shows the annual influenza-attributed mortality per age category, as well as the overall annual influenza mortality and years-of-life-lost. For the pandemic, a total of 612 deaths (95% CI 266–958) were attributed to influenza, corresponding to 3.70 deaths (1.61–5.79) per 100,000 population. This is much lower than the mean 1,956 annual deaths for seasonal influenza (Table 2; corresponding to 11.83 deaths per 100,000 pop.); however, the number of years-of-life-lost for the pandemic was estimated at 15,845, which comes close to the mean 17,908 years-of-life-lost for seasonal influenza (although for 6 out of 10 seasonal epidemics more years-of-life-lost were estimated than for the pandemic, Table 2).

**Table 2:** Annual influenza-attributed deaths and years-of-life-lost (YLL) by age category.

		Number of influenza-attributed deaths							YLL
		By age category						Total	
		0–4	5–24	25–44	45–64	65–74	≥75		
**Seasonal influenza**	**1999/2000**	4 (0–11)	0 (0–2)	13 (0–36)	60 (14–106)	291 (182–400)	2,310 (1,891–2,730)	2,679 (2,234–3,108)	19,512
	**2000/2001**	37 (29–45)	38 (28–48)	12 (0–37)	0 (0–0)	0 (0–13)	0 (0–279)	87 (0–183)	5,906
	**2001/2002**	19 (10–29)	22 (7–37)	30 (0–70)	142 (76–209)	222 (73–372)	2,257 (1,673–2,840)	2,693 (2,085–3,301)	23,650
	**2002/2003**	0 (0–0)	21 (12–31)	10 (0–41)	176 (128–224)	328 (230–426)	1,716 (1,361–2,070)	2,251 (1,859–2,604)	20,614
	**2003/2004**	24 (18–30)	3 (0–13)	62 (36–89)	233 (193–273)	393 (314–472)	2,232 (1,920–2,543)	2,947 (2,622–3,271)	28,933
	**2004/2005**	29 (24–34)	20 (7–33)	41 (14–68)	170 (117–222)	205 (123–286)	3,170 (2,829–3,511)	3,634 (3,278–3,990)	30,243
	**2005/2006**	15 (8–22)	15 (0–31)	0 (0–21)	8 (0–73)	0 (0–0)	1,096 (661–1,532)	1,134 (435–1,341)	8,388
	**2006/2007**	0 (0–0)	0 (0–13)	15 (0–40)	0 (0–0)	0 (0–19)	415 (105–725)	430 (0–619)	2,942
	**2007/2008**	26 (18–33)	13 (0–28)	62 (36–88)	111 (38–185)	0 (0–12)	396 (69–723)	608 (182–879)	10,589
	**2008/2009**	10 (0–21)	12 (0–25)	60 (29–91)	187 (94–279)	397 (303–491)	2,434 (1,975–2,893)	3,100 (2,622–3,579)	28,298
	**Average over 10 seasonal epidemics (1999/2009)**	16	15	31	109	184	1,603	1,956	17,908
**Pandemic**	**2009/2010**	77 (61–93)	30 (13–48)	29 (0–58)	46 (0–121)	313 (232–394)	116 (0–442)	612 (266–958)	15,845

The 95% confidence intervals for the influenza-attributed deaths by age category are given in brackets behind the point estimates. In the last two columns on the right-side of the table for each year in the study period the total number of influenza-attributed deaths is given as well as the total years-of-life-lost attributed to influenza.

**Figure 2:**
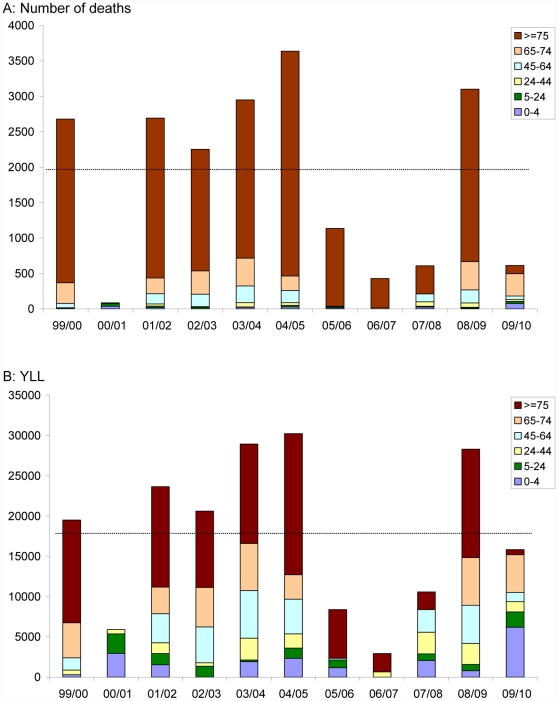
Total (a) number of deaths and (b) years-of-life-lost (YLL) attributed to influenza by age category for the pandemic (2009/2010) and all other years included in this study (1999–2009). The horizontal black dotted lines indicate the average total number of deaths and years-of-life-lost for the 10 seasonal epidemics in 1999–2009.

Figure 2 further illustrates the difference in impact by number of deaths (Figure 2a) or years-of-life-lost (Figure 2b). For the 0–4 yrs of age – the category with the highest residual life expectancy – the number of influenza-attributed deaths during the pandemic was higher than in any seasonal epidemic in the study period (Table 2 and Figure 2a). For the age categories 5–24, 25–44, 45–64 and 65–74 yrs of age, some seasonal epidemics show higher and others lower numbers of deaths in these age categories than during the pandemic. Finally, the ≥75 yrs of age have a far below average number of deaths during the pandemic. The numbers of pandemic deaths by age category in Table 2 and Figure 2a correspond to a population rate of 8.30 (95% CI 6.58–10.03) pandemic deaths per 100,000 in 0–4 yrs of age; 0.76 (0.32–1.19) in 5–24 yrs; 0.65 (0–1.29) in 25–44 yrs; 1.00 (0–2.64) in 45–64 yrs; 22.79(16.92–28.67) in 65–74 yrs; and 10.27 (0–39.04) in ≥75 yrs of age.

Sensitivity analysis showed that RSV and hot/cold weather only minimally influenced the influenza-attributed mortality. Replacing ILI-incidence by influenza detections in the models did not change the estimates much either. When replacing all-cause mortality by respiratory/cardiovascular or pneumonia/influenza deaths, the estimates for pandemic and seasonal influenza deaths were much lower; but pandemic mortality was consistently estimated lower than during an average seasonal influenza epidemic, with a relatively low impact in the elderly combined with a relatively high impact in the youngest age category. See Appendix S2 for further details.

Because of the increased number of influenza-attributed deaths in the 0–4 yrs of age during the pandemic year, we further explored the distribution of age and reported cause of death in this age category. After excluding neonatal deaths, deaths at <1 yrs of age occurred relatively more often during the pandemic than in earlier seasonal influenza years (right-sided Fisher's exact tests for 2×2 tables; P≤0.022), but to a lesser extent this was also observed for the seasonal epidemic in 2000/2001. Among the reported causes of death, cardiomyopathy (International Classification of Diseases, 10^th^ revision, ICD-10 code I42) and spinal muscular atrophy (ICD-10 code G12.9) were overrepresented during the pandemic exclusively (P≤0.002 and P≤0.012 respectively). Intracerebral haemorrhage in neonates was also disproportionately high during the pandemic (ICD-10 P52.4; P≤0.004), but also in 2004/2005 although then only borderline significant. The two other causes of death that showed elevations (fetus/newborn with necrotizing enterocolitis and fetus/newborn affected by multiple pregnancy, ICD-10 P77 and P01.5) seemed unlikely to be related to the pandemic, since the first had been increasing since 2007 and the second showed elevations for two other years in the study. See Appendix S3 for further details.

## Discussion

By comparing mortality estimates of the 2009 pandemic with those for seasonal influenza, we found that deaths in young children disproportionately contributed to the number of pandemic deaths compared to seasonal influenza. However, the overall pandemic impact was only moderate with the total years-of-life-lost somewhat below but close to the average of seasonal influenza, and the total number of deaths far below average. This limited impact – despite disproportionate mortality in young children – was mainly due to low mortality in the elderly (≥75 yrs of age) compared to most seasonal epidemics.

### Comparing pandemic vs seasonal influenza mortality

We were able to compare pandemic to seasonal influenza mortality by using the same statistical methodology and data types for both pandemic and seasonal influenza years. Our use of annual instead of constant ILI-coefficients made it possible to estimate the influenza-attributed mortality per year, adjusted for shifts in the association of ILI-incidence and mortality.

We found a large range of total numbers of deaths and years-of-life-lost for the 10 seasonal epidemics. In 2003/2004 the influenza A/Fujian/411/02(H3N2) drift variant emerged and Fujian-like H3N2 viruses remained the dominant subtype in the next 3 years [22]; not surprisingly, we observed high seasonal influenza mortality in 2003/2004 and 2004/2005. However, over the following 2 years - with still Fujian-like H3N2 viruses as the dominant subtype - we observed a sharp decline in influenza-attributed mortality (Table 2); this decrease in mortality impact may be due to increasing population immunity over the years by successive natural infection and vaccination with A(H3N2) viruses of similar antigenicity. For the other seasonal epidemics, the years with H3N2 subtypes as dominant viruses (1999/2000, 2001/2002, 2008/2009) showed relatively high mortality, whereas the H1N1 seasons (2000/2001, 2007/2008) showed relatively low mortality, which seems in line with the fact that mortality levels tend to be lower in seasons with predominantly A(H1) strains than in A(H3) seasons [23], [24]. The mortality during the two H1N1 seasonal epidemics appears to be especially low in older age groups, as also observed for the 2009 pandemic; this may be due to relatively high (memory) immunity for H1N1 viruses in people born before 1957, induced by viruses descending from the 1918/1919 pandemic virus, as has been reported for the 2009 pandemic [25].

### Disproportionate mortality in young children

For the 0–4 yrs of age, we estimated 77 pandemic deaths, which is higher than for any seasonal influenza epidemic in the study. Remarkably, in the Netherlands only 5 laboratory-confirmed deaths in 0–4 yrs of age were reported during the pandemic, including one child <1 yrs of age with a medical condition [26], [27]. This low number of laboratory-confirmed pediatric deaths, compared to the 77 estimated pandemic deaths in this study, raises the question whether pediatric deaths due to pandemic influenza may have gone unrecognized. Alternatively, the high number of influenza-attributed deaths in young children during the pandemic may be spurious, due to a rise in deaths by other causes at the same time. However, to our knowledge there were no other unusual competing events or epidemics that may explain such an increase in deaths in young children.

The higher proportion of deaths in <1 yrs of age (excluding neonates) that we observed, suggests that a disproportionate fraction of the 77 estimated influenza-attributed deaths may have occurred in infants <1 yrs of age who may be less likely to show sufficiently specific symptoms of influenza infection to prompt requests for influenza laboratory diagnostics. In England and Argentina, high mortality rates in infants have been reported among pediatric deaths due to laboratory-confirmed influenza A(H1N1) 2009 infection [28], [29]. For Australia and the US, studies that attributed all-cause mortality to influenza did not report many deaths in infants [9], [10]; for the Australian study, infant deaths were subsumed under the 0–19 yrs of age category and thus excess infant mortality may have been unrecognized; in the US study, age-specific mortality estimates were extrapolated from the age distribution of laboratory-confirmed deaths, so mortality in infants would only have been picked up after laboratory confirmation. However, in two recent studies also attributing age stratified all-cause mortality to influenza – including 0–4 yrs of age as in our study – no increased mortality in 0–4 yrs of age was observed for Denmark and Mexico [30], [31]; estimates for several other age categories were also different, and all-age pandemic mortality was estimated considerably higher for Mexico; this suggests heterogeneity in pandemic mortality and its age distribution between countries and regions, although different model choices may partly account for the reported differences.

Our observation of 8 deaths by cardiomyopathy during the pandemic in the 0–4 yrs of age, and 5 deaths by spinal muscular atrophy – even if confirmed to be related to pandemic influenza infection – cannot quantitatively explain our estimate of 77 pandemic deaths. Nevertheless, cardiomyopathy and spinal muscular atrophy are not unlikely to be related to influenza. Heart disease is a known complication of influenza and has also been specifically reported for pandemic influenza A(H1N1) 2009 [32], [33], [34], [35]; the observed 8 cases of cardiomyopathy may well represent more deaths by cardiomyopathy in young children, since autopsy after an unexplained death is not standard. Spinal muscular atrophy is a neurological condition that can lead to death from a respiratory infection during early childhood [36], [37]; the disproportionate level of spinal muscular atrophy related deaths seems to be in line with the reported 9 deaths with neurological disorders out of a total of 65 laboratory-confirmed pandemic deaths [27]. To what extent the disproportionate number of intracerebral haemorrhage in neonates may be related to pandemic influenza seems more speculative, but fluctuating blood pressure by coughing caused by influenza might be an explanation [38].

### Mortality vs morbidity

Unlike two earlier Dutch studies on the impact of the pandemic [8], [39], we ignored the impact of influenza morbidity. In one of these studies, the pandemic was compared to seasonal influenza morbidity based on the GP-ILI-incidence [8] – the pandemic ILI-incidence appeared to be most marked among 5–14 yrs of age – but no analyses on severity of infection or mortality were performed.

In the other Dutch study [39], we estimated the total disease burden by pandemic mortality and morbidity in the Netherlands. This study had some limitations when comparing the pandemic with seasonal influenza. Firstly, the number of years-of-life-lost during the pandemic – which covered around 40% of the total estimated pandemic disease burden – was restricted to laboratory-confirmed deaths reported and was thus probably underestimated. Secondly, in this earlier study we assumed the same severity weight per influenza case for seasonal and pandemic influenza; this assumption may thus lead to over- or underestimation of the disease burden due to pandemic influenza morbidity. To avoid this issue in our current study we only included mortality as a measure of influenza severity, since death as a final outcome cannot be less or more severe. It can be expected though that less or more severe influenza morbidity will be reflected in lower or respectively higher mortality and consequently is at least partly captured by our study results.

### Limitations

Several methods can be used to estimate influenza deaths from mortality data, like annual regression methods [40], [41], weekly or monthly regression models with mortality attributed to influenza using a seasonal baseline [9], [10], or using the association of mortality with measured influenza activity [42]. Since we had consistent weekly data on influenza activity and age-specific mortality available for both the pandemic and the 10 preceding seasonal epidemics, we chose for regression models associating age-specific weekly mortality to influenza activity. In such models, pathogens or factors other than influenza can still bias the modeled association of mortality and influenza activity. To minimize this possibility, we considered pathogens other than influenza for inclusion in our models as well. However, during the pandemic several pathogens other than influenza appeared to be detected disproportionately often, probably due to increased diagnostic testing to exclude H1N1 infection. In addition, some pathogens (e.g. rhinovirus) showed an increasing trend of weekly counts in recent years, possibly reflecting the implementation of new diagnostic tests. As artificial increases in laboratory counts can bias the modeled association of pathogens with mortality, we did not include the data of most pathogens. The only pathogen we did include was Respiratory Syncytial virus (RSV); RSV is known to cause severe morbidity, especially in young children but also in adults [14], [15], and thus could possibly affect the estimates for influenza-associated mortality. We did, however, adjust the RSV data for the supposed artificial increase during the pandemic (see methods section). In the years before the pandemic, the annual number of RSV detections was stable, so biases by new diagnostic tests seemed unlikely.

Nevertheless, our estimates for influenza mortality can be affected by the way we chose to model the association of mortality with influenza activity in the population; another study [43] resulted in different influenza mortality estimates for some years compared to the current study. Several explanatory variables had been different in that other study, so we decided to explore the impact of the selected explanatory variables on the results of the current study. We found that replacing the ILI-incidence in the models by influenza detections resulted in similar mortality estimates for pandemic and seasonal influenza (Appendix S2). However, the association of mortality and influenza detections may have been biased due to enhanced testing during the peak of the pandemic. Secondly, we found that the influence of RSV and the hot/cold weather variables on the estimated influenza-attributed mortality was minimal (Appendix S2). Our use of seasonal variables and autocorrelation in the models should have corrected for mortality caused by pathogens we did not include, and other seasonal or possibly transient causes of mortality.

Some consider mortality categories such as pneumonia/influenza and respiratory/cardiovascular deaths more specific for influenza than all-cause mortality [9], [40]. On the other hand, such mortality categories are less sensitive for influenza mortality, as deaths due to influenza can be coded in other categories as well. Also, differences in coding practice in time or between countries have no influence on all-cause mortality. Nevertheless, as a validation of our results we showed that using pneumonia/influenza and respiratory/cardiovascular deaths in our models resulted in the same pattern of low pandemic mortality compared to seasonal influenza, with relatively high pandemic mortality in the youngest age group.

Finally, the residual life expectancy that we used to estimate the years-of-life-lost may be lower for people with underlying medical conditions (who are more likely to die from influenza; the so called “harvesting effect”), leading to a potential overestimation of years-of-life-lost. In an earlier study on laboratory-confirmed pandemic deaths, modifying the life expectancy based on reported underlying disease resulted in a reduction of years-of-life-lost by over a third [39]. Nevertheless, comparing the pandemic years-of-life-lost with seasonal epidemics seems still appropriate, as also during seasonal epidemics severe morbidity and thus mortality will likely be higher in people with underlying medical conditions; this is illustrated by a study on pediatric influenza cases in the US, where a similar percentage of patients with underlying medical conditions was reported for severe morbidity by seasonal and pandemic influenza [44].

### Conclusion

We found that the pandemic had an overall moderate impact on mortality compared to the 10 preceding seasonal epidemics; disproportionate mortality in young children was observed, as opposed to low mortality in ≥75 yrs of age. This resulted in a far below average total number of pandemic deaths compared to seasonal influenza, but a total number of years-of-life-lost only somewhat below average. To assess the impact of future pandemics, attributing age-specific all-cause mortality to influenza should be preferred above comparing laboratory-confirmed deaths to historical estimates for seasonal influenza. For other countries and continents, pandemic mortality should be estimated relative to seasonal influenza as well, to assess the worldwide impact of the 2009 pandemic.

## Supporting Information

Figure S1 Weekly all-cause mortality by age category and ILI-incidence in time.
(DOC)

Appendix S1 Details on regression models.
(DOC)

Appendix S2 Sensitivity analysis.
(DOC)

Appendix S3 Deaths in 0–4 yrs of age during the pandemic compared to seasonal influenza; distribution of age and causes of death.
(DOC)
