# Supplementary material for: Comparing Pandemic to Seasonal Influenza Mortality: Moderate Impact Overall but High Mortality in Young Children
Source: PLoS One. 2012 Feb 3;7(2):e31197. doi: 10.1371/journal.pone.0031197 (PMC3272034; doi:10.1371/journal.pone.0031197)
Supplement: Figure S1 — Weekly all-cause mortality by age category and ILI-incidence in time. (DOC) [file pone.0031197.s001.doc]

**Figure S1: Weekly all-cause mortality by age category and ILI-incidence in time.**

The grey-dotted vertical lines indicate the episodes -3/+3 weeks around influenza epidemics (defined as two or more successive weeks that the overall weekly ILI-incidence was above the threshold for influenza epidemics; i.e. 5.1 per 10,000 population [11]).
