# Supplementary material for: Comparing Pandemic to Seasonal Influenza Mortality: Moderate Impact Overall but High Mortality in Young Children
Source: PLoS One. 2012 Feb 3;7(2):e31197. doi: 10.1371/journal.pone.0031197 (PMC3272034; doi:10.1371/journal.pone.0031197)
Supplement: Appendix S1 — Details on regression models. (DOC) [file pone.0031197.s002.doc]

**Appendix S1: Details on regression models**

We used an additive Poisson model [1] for the weekly death counts in each age category:

*C(t) ~ Poisson( b_0_ + b_1_ t +  b_2_ ILI(t+n_1_) + b_3_ RSV(t+n_2_) + b_4_ T_L_(t) + b_5_T_H_ (t)+ sine + cosine)*

*C(t)* the death counts at time t

*t*  time in weeks over the whole study period (1-574 weeks)

*b_0_* regression coefficient describing constant basic mortality level

*b_1_-b_5_* regression coefficients

*ILI(t)* overall (all ages together) ILI-incidence at time t *

*RSV(t)* counts of RSV at time t detected in the laboratory

*n_i_* lag time in weeks for ILI or RSV, -4 ≤ n_i_ ≤ +2 weeks.

*T_L_(t)* Cold weather variable defined as the (positive) number of degrees Celsius below 0 ^o^C of the average weekly temperature at time t

*T_H_(t)* Hot weather variable defined as the (positive) number of degrees Celsius above 17 ^o^C of the average weekly temperature at time t

*sine* sine terms to describe seasonally varying mortality level sine(k2πweek/52), k=1,2,3,4

*cosine* cosine terms to describe seasonally varying mortality level

cosine(k2πweek/52), k=1,2,3,4

* Only ILI data in and around influenza seasons was included - ILI data in all other weeks was truncated at zero in the regression models. In Figure 1a-b and Figure S1 the episodes are indicated that the ILI incidence was included in the models.

We implemented this model in SAS version 9.2 (SAS Institute Inc., Cary, NC, USA) by constructing GEE regression models with a Poisson distribution and an identity link [1,2]. For this, an additive model for the incidence was multiplied by the population size to obtain a model for the counts, as described in [3]. Thus we could model proportional associations between the explanatory variables and the Poisson distributed mortality counts variables, taking into account changes in population size.

We initially built a generalized linear model with a Poisson distribution and an identity link. To correct for mortality due to factors other than influenza, we used a constant basic mortality level plus sine and cosine variables (sine(k2πweek/52) and cosine(k2πweek/52), k=1,2,3,4) correcting for seasonal variation. We also added the hot and cold weather variables to correct for mortality due to hot and/or cold weather conditions. For the 5-24 yrs of age the cold weather variable was removed again because negative associations were estimated by the model. We then used a forward stepwise regression approach to add the ILI-incidence and the RSV counts to the model, selecting the lagged ILI or RSV variable that contributed most to the model fit (-4 up to +2 week lags were considered for inclusion; e.g., in step 1, ILI was included with a 1-week lag if that exhibited a better model fit than all other ILI/RSV-lag combinations, assessed with Akaike’s information criterion [4]). We included lagged GP–ILI-incidence and the RSV counts in the model only once.

When first building the models, the ILI and other model coefficients were assumed to be constant in time. As earlier described in [3], we then replaced the constant regression coefficient for ILI by different regression coefficients for separate years (between July 1^st^-June 30^st^). The annual regression coefficients for ILI then describe the association between mortality and incidence of ILI by year. Finally, we used GEEs [1] to correct the model outcomes for autocorrelation between observations. For the GEEs we defined each influenza year as a subject (July 1^st^-June 30^th^).

Two years in the study period contained 53 weeks, whereas the sine and cosine variables assume a maximum of 52 weeks per year. In the regression models, we therefore smoothed the sine and cosine curves by using week number 52.5 instead of 53 for those two weeks. Note that for graphic presentation of the results, we transformed the count model outcomes into incidences, see Figure 1a-b. To be able to compare the annual estimates for influenza-attributed mortality in each age category between different years, we used 2009 as a reference and scaled all annual mortality counts to the 2009 population.

1. Boshuizen HC, Feskens EJ (2010) Fitting additive Poisson models. Epidemiol Perspect Innov 7: 4.

2. Liang KY, Zeger SL (1986) Longitudinal data analysis using generalized linear models. Biometrika 73: 13-22.

3. van den Wijngaard CC, van Asten L, Meijer A, van Pelt W, Nagelkerke NJ, et al. (2010) Detection of Excess Influenza Severity: Associating Respiratory Hospitalization and Mortality Data With Reports of Influenza-Like Illness by Primary Care Physicians. Am J Public Health 100: 2248-2254.

4. Akaike H (1974) A new look at statistical model identification. IEEE Transactions on automatic Control AU-19: 716-723.
