# Supplementary material for: Comparing Pandemic to Seasonal Influenza Mortality: Moderate Impact Overall but High Mortality in Young Children
Source: PLoS One. 2012 Feb 3;7(2):e31197. doi: 10.1371/journal.pone.0031197 (PMC3272034; doi:10.1371/journal.pone.0031197)
Supplement: Appendix S2 — Sensitivity analysis. (DOC) [file pone.0031197.s003.doc]

**Appendix S2: Sensitivity analysis**

We performed a sensitivity analysis to evaluate the impact of our model choices on the influenza-attributed mortality estimates. For this we evaluated the following 6 models:

- Model 1: the full model as described in Appendix S1, with ILI as explanatory variable to attribute all-cause mortality to influenza, and hot/cold weather and RSV as additional explanatory variables.
- Model 2: without hot/cold weather as explanatory variables.
- Model 3: without RSV as explanatory variable.
- Model 4: with influenza detections (instead of ILI) as explanatory variable to attribute all-cause mortality to influenza
- Model 5: with respiratory and cardiovascular (ICD-10 codes J00-J99 and I00-I99) deaths as explained variable (instead of all-cause mortality)
- Model 6: with pneumonia/influenza (J09-J18) deaths as explained variable (instead of of all-cause mortality)

The models 5 and 6 with mortality by death cause could not be fitted in the youngest age categories due to low numbers. Therefore we present all results of the sensitivity analysis with 0-44 yrs of age instead of 0-4, 5-24 and 25-44 yrs of age.

*Removing hot/cold weather or RSV from the model (model 2 and 3)*

We evaluated to what extent the estimates on influenza-attributed mortality changed by removing explanatory variables from the regression models. Figure S2a-b shows that the total number of deaths remained stable after excluding RSV and hot/cold weather variables from the model with annual ILI-coefficients (model 1 compared to models 2-3); both for an average seasonal epidemic as for the pandemic the estimates only minimally changed. The number of deaths by age category for an average seasonal influenza and the pandemic remained quite stable as well; only the number of deaths in ≥75 yrs of age during the pandemic dropped to zero when excluding RSV from the model.

*Influenza detections as an alternative measure of influenza activity (model 4)*

Influenza detections in the laboratory can be an alternative for ILI-incidence as a measure of influenza activity in the models. To evaluate this alternative, we summed all weekly influenza A and B detections, as reported by a routine laboratory surveillance system, and included them in the models. Similar as in model 1, we only included deaths attributed to influenza detections within a -3/+3 week window around influenza epidemic episodes. Figure S2 shows that the average number of deaths per seasonal epidemic in the study was estimated roughly the same when using influenza detections (2,098 vs 1,956 with ILI-incidence); for the pandemic the estimate was somewhat lower with influenza detections (521 vs 612 with ILI-incidence). The number of deaths by age category for an average seasonal influenza remained quite stable as well, although somewhat higher in most age categories; for the pandemic the estimated numbers of deaths was lower in most age categories, but higher in ≥75 yrs of age.

*Mortality by death cause as explained variables (model 5 and 6)*

We evaluated the impact on our results of using pneumonia/influenza or respiratory/cardiovascular deaths as explained variable instead of all-cause mortality data. As expected influenza-attributed mortality estimates were lower for seasonal influenza when using respiratory/cardiovascular deaths, and even lower when using pneumonia/influenza deaths (Figure S2a-b). For the pandemic, total numbers of influenza-attributed deaths were again lower compared to all-cause mortality, but in the ≥75 yrs of age the number of influenza-attributed deaths was highest in pneumonia/influenza deaths.

In all models used in the sensitivity analysis, total pandemic mortality was consistently much lower than during an average seasonal influenza epidemic; this was due to a relatively low impact in the elderly, but at the same time a relatively high impact in the youngest age category was observed.

**Figure S2. Mortality for (a) an average seasonal epidemic (1999-2009) versus (b) the pandemic (2009/2010).**
