# Supplementary material for: Comparing Pandemic to Seasonal Influenza Mortality: Moderate Impact Overall but High Mortality in Young Children
Source: PLoS One. 2012 Feb 3;7(2):e31197. doi: 10.1371/journal.pone.0031197 (PMC3272034; doi:10.1371/journal.pone.0031197)
Supplement: Appendix S3 — Deaths in 0–4 yrs of age during the pandemic compared to seasonal influenza; distribution of age and causes of death. (DOC) [file pone.0031197.s004.doc]

**Appendix S3: Deaths in 0-4 yrs of age during the pandemic compared to seasonal influenza; distribution of age and causes of death.**

Because of the high number of influenza-attributed deaths in the 0-4 yrs of age during the pandemic year, we further explored the distribution of age and causes of death in this age category compared to other years.

Figure S3 shows the two-step criteria that we used to evaluate whether disproportionate deaths in <1yrs of age and/or deaths by specific causes might be related to the high number of ILI-attributed deaths in the 0-4 yrs of age during the pandemic. In step 1, we assessed whether deaths in <1yrs of age and/or primary causes of death among the 0-4 yrs of age were disproportionately higher during the pandemic than in earlier years. For this, we compared the pandemic with earlier seasonal influenza epidemics. However, the pandemic occurred earlier in the year than most seasonal influenza epidemics, which could lead to biased results since we could not exclude that age and/or cause of death vary somewhat by time of the year. Therefore, we also compared the pandemic with the periods between the same dates as the pandemic in earlier years. Thus found disproportionate levels of <1yrs of age and/or specific causes of death were considered as possibly relevant if they were significantly higher both compared to (1) the periods with the same dates and (2) the seasonal epidemics in earlier years. For the causes of death, of which some had very low counts, we only evaluated ICD-10-codes (International Classification of Diseases, 10^th^ revision) with ≥5 deaths during the pandemic. If we thus found no relevant 4-digit level ICD-10-codes (e.g. A00.1), we also evaluated the corresponding 3-digit level codes (e.g. A00).

Finally in step 2 (Figure S3), we verified whether thus found disproportionate levels of specific causes of death and/or <1yrs of age during the pandemic also occurred in other years in the study. We did this by using the same analyses as for the pandemic, but then compared each seasonal epidemic to the other years with seasonal epidemics in the study.

In Table S3 it can be seen that mortality in the <1yrs of age only showed a higher proportion during the pandemic if compared to the episodes of seasonal epidemics, and not if compared to the episodes between the same dates in earlier years. However, after excluding neonatal deaths, the <1yrs of age showed a higher proportion during the pandemic according to the criteria in Figure S3. In 2000/2001 the <1yrs of age without neonates also showed a higher proportion, although only borderline significant. The other age groups that we tested for showed no higher proportions during the pandemic. Table S3 also shows that we observed higher proportions for the causes of death cardiomyopathy (I42) and spinal muscular atrophy (G12.9) exclusively during the pandemic. Intracerebral (nontraumatic) haemorrhage of fetus and newborn (P52.4) showed a higher proportion in 2004/2005 as well, although only borderline significant. The two remaining causes of death in Table S3 seemed unlikely to be specifically related to the pandemic, since one showed an increasing trend since 2007 (Necrotizing enterocolitis of fetus and newborn; P77) and the other showed higher proportions for two other years in the study (Fetus and newborn affected by multiple pregnancy; P01.5).

**Figure S3. Assessing differences in age distribution and/or causes of death among 0-4 yrs of age during the pandemic compared to earlier years in this study (1999-2009).**

**STEP 1**

**STEP 2**

**Are the proportions of age groups and/or specific causes of death different for the pandemic than for earlier years within the 0-4 yrs of age deaths?**

**Proportion of specific age groups***

- if an age group has a significantly higher proportion** when comparing the pandemic with:
  - seasonal epidemics in earlier years***

AND

- - the same episodes in earlier years ****

**Proportion of specific causes of death**

- if a specific death cause has a significantly higher proportion when comparing the pandemic with:
  - seasonal epidemics in earlier years***

AND

- - the same episodes in earlier years ****

AND

- if a specific death cause has >= 5 cases during the pandemic

**How unique are these findings during the pandemic compared to other years?**

- We assessed whether other years in the study also showed similar higher proportions. Each year was compared to the rest of the years with the same approach as in step 1.*****

* We performed the analyses for ≤1 week (perinatal), ≤1month (neonatal), <1 yrs and 1-4 yrs of age.

** As we evaluated by the right-sided Fisher’s exact test for 2x2 tables (P≤0.05).

*** Influenza epidemic episodes were defined as -3/+3 weeks around successive weeks with an ILI-incidence >5.1 per 10,000 population.

**** Each year we used the same dates as we defined for the pandemic in this study; i.e. September 16th until January 3rd.

***** The 2009/2010 pandemic year was excluded. Only causes of death with higher levels during the pandemic were evaluated.

**Table S3 Test results for higher proportions of specific age groups and/or causes of death** during the pandemic compared to earlier years in the study, as evaluated by right-sided Fisher’s exact tests for 2x2 tables.

|  |  |  | **Higher proportion during pandemic***** | |  |
| --- | --- | --- | --- | --- | --- |
|  |  | **Number of deaths out of all 0-4 yrs of age deaths during the pandemic*** | **As compared with seasonal epidemics in earlier years** | **As compared with the same episodes in earlier years** | **Did specific seasonal epidemics in the study show similar results?** |
| **Age** | Perinatal deaths** | 136 out of 288 | P=0.518 (NS) | P=0.713 (NS) | n/a**** |
|  | Neonatal deaths** | 179 out of 288 | P=0.424 (NS) | P=0.782 (NS) | n/a**** |
|  | <1yrs of age | 250 out of 288 | P=0.016 | P=0.103 (NS) | n/a**** |
|  | **<1yrs of age, excluding neonatal deaths**** | 71 out of 109 | P=0.007 | P=0.022 | In 2000/2001 a higher proportion was observed as compared to other seasonal epidemics (62 out of 102 deaths; p=0.048) |
|  | 1-4 yrs of age | 38 out of 288 | P=0.990 (NS) | P=0.926 (NS) | n/a**** |
|  |  |  |  |  |  |
| **Primary causes of death** | **Cardiomyopathy (I42)** | 8 out of 277 | P=0.002 | P=0.001 | Higher proportion only observed during pandemic |
|  | **Spinal muscular atrophy, unspecified (G12.9)** | 5 out of 277 | P=0.012 | P=0.002 | Higher proportion only observed during pandemic |
|  | Fetus and newborn affected by multiple pregnancy (P01.5) | 5 out of 277 | P=0.020 | P=0.040 | In 2005/2006 a higher proportion was observed as compared to the same episodes in other years *and* other seasonal epidemics (5 out of 350 deaths; p=0.020 and p=0.018)  And in 2007/2008 a higher proportion was observed as compared to the same episodes in other years (although only 3 out of 206 deaths; p=0.042) |
|  | **Intracerebral (nontraumatic) haemorrhage of fetus and newborn (P52.4)** | 10 out of 277 | P=0.004 | P<0.001 | In 2004/2005 a higher proportion was observed as compared to the same episodes in other years (7 out of 335 deaths; p=0.045) |
|  | Necrotizing enterocolitis of fetus and newborn (P77) | 11 out of 277 | P=0.010 | P=0.022 | In 2007/2008 a higher proportion was observed as compared to the same episodes in other years *and* other seasonal epidemics. (7 out of 206 deaths; p=0.011 and p=0.044) This death cause seemed to show an increasing trend since 2007. |

NS=non-significant

*The 2010 death cause data were not available yet at the time of analyses, so causes of death from January 1^st^ -3^rd^ 2010 were not included.

**Perinatal deaths defined as all deaths within ≤1 week of age; Neonatal deaths defined as all deaths with ≤1 month of age.

*** As we evaluated by the right-sided Fisher’s exact test for 2x2 tables (P≤0.05).

****Not applicable, since no relevant higher proportions of deaths within these age groups were observed during the pandemic, according to the criteria in Figure S3.
